# Closing the Loop: A Re-Audit of Local Clinical Governance in Mental Health Services Across the London and South Region (2025-2026)

**DOI:** 10.1192/bjo.2026.11830

**Published:** 2026-06-30

**Authors:** Gayathri Rangith

**Affiliations:** Cygnet Health Care, Oldbury, United Kingdom

## Abstract

**Aims:**

Local Clinical Governance (LCG) provides a structured framework through which healthcare organisations are accountable for continuously improving service quality and patient safety. A multi-site audit conducted between September and December 2024 across mental health hospitals identified variability in leadership engagement, multidisciplinary team (MDT) attendance, safety reporting, and documentation of clinical effectiveness. This re-audit aimed to evaluate the impact of targeted quality improvement interventions implemented following the initial audit, assessing whether governance standards improved across participating sites.

**Methods:**

A closed-loop re-audit was conducted between September and December 2025 across 27 mental health hospital sites. Data were collected from Local Clinical Governance meeting minutes using a structured audit tool aligned with national clinical governance standards and the STEELL agenda (Safety, Training, Effectiveness, Experience, Leadership, Lessons Learned). Key domains assessed included meeting leadership and attendance,pharmacy involvement, safety and incident reporting, care-plan and GAP compliance, dissemination of lessons learnt, and patient and carer engagement. Findings were compared with the baseline audit conducted during the same months in 2024.

**Results:**

The re-audit demonstrated measurable improvements across multiple governance domains. Leadership accountability improved following the introduction of mandatory co-chairing of LCG meetings by Medical Directors and Hospital Managers, with leave planning structured around monthly governance meetings. Pharmacy attendance improved, strengthening oversight of medication safety. Enhanced contraband confiscation processes and improved dissemination of lessons learnt were observed across sites. Clinical effectiveness improved, with GAP consistently completed at admission and discharge, and care-plan triangulation optimised through integration of risk assessments, clinical documentation, and governance review. Patient and carer engagement improved through structured carer events, facilitating better communication between families and clinical teams. Some inter-site variability persisted, highlighting areas for continued improvement.

**Conclusion:**

This closed-loop re-audit demonstrates that targeted, leadership-driven quality improvement interventions can significantly enhance Local Clinical Governance processes across mental health services. Strengthening leadership accountability, MDT engagement, and systematic learning contributes to improved safety, clinical effectiveness, and patient and carer experience. These findings highlight the value of re-audit cycles in embedding sustainable governance improvements and offer transferable learning for other mental health providers.

